# Supplementary material for: Promoting the adoption of best practices and standards to enhance quality and reproducibility of stem cell research
Source: Stem Cell Reports. 2025 Jun 12;20(7):102531. doi: 10.1016/j.stemcr.2025.102531 (PMC12277813; doi:10.1016/j.stemcr.2025.102531)
Supplement: Document S1. Table S1 [file mmc1.pdf]

Stem Cell Reports, Volume *20*

Supplemental Information

Promoting the adoption of best practices and standards to enhance quality and reproducibility of stem cell research

Lucia Selfa Aspiroz, Milena Mennecozzi, Laura Batlle, Barbara Corneo, Lyn Healy, Mark Kotter, Andreas Kurtz, Tenneille E. Ludwig, Christine Mummery, Martin Pera, Glyn N. Stacey, Carlos A. Tristan, and Maurice Whelan

Supplemental Information

Supplementary Table 1: ISO Biotechnology Standards relevant to stem cell culture

General topic	ISO reference	Status
Analytical methods (cell line testing and characterisation)	ISO/TS 23511:2023 - General requirements and considerations for cell line authentication	Published (May 2023)
	ISO/CD 23511 - General requirements and considerations for cell line authentication	Under development, will replace ISO/TS 23511:2023
	ISO 24479:2024 - Cellular morphological analysis – General requirements and considerations for cell morphometry to quantify cell morphological features	Published (October 2024)
	ISO/DIS 8934-1 - Cell viability analytical methods- Part 1: General requirements and considerations	Under development
	ISO 20391:2018 - Cell counting - Part 1: General guidance on cell counting methods	Published (January 2018)
	ISO/CD 20391-1 - Cell counting - Part 1: General guidance on cell counting methods	Under development, will replace ISO 20391-1:2018
	ISO 20391-2:2019 - Cell counting - Part 2: Experimental design and statistical analysis to quantify counting methods	Published (August 2019)
	ISO 24190:2023 - Analytical methods – Risk-based approach for method selection and validation for rapid microbial detection in bioprocesses	Published (May 2023)
	ISO/CD TR 4752 - Inventory of methods for detection of microbiological contamination in mammalian cell culture	Under development
Biobanking	ISO 21709:2020 - Biobanking – Process and quality requirements for establishment, maintenance and characterisation of mammalian cell lines	Published with an amendment ISO 21709:2020/Amd 1:2021
	ISO 24603:2022 - Biobanking - Requirements for human and mouse pluripotent stem cells	Published (August 2022)

	ISO 18162:2024 - Biobanking - Requirements for human neural stem cells derived from pluripotent stem cells	Published (December 2024)
	ISO/DIS 20012 - Biobanking - Requirements for human natural Killer cells derived from pluripotent stem cells	Under development
	ISO/TS 22859:2022 - Biobanking – Requirements for human mesenchymal stromal cells derived from umbilical cord tissue	Published (July 2022)
	ISO 24651:2022 - Biobanking - Requirements for human mesenchymal stromal cells derived from bone marrow	Published (August 2022)
Data interoperability for stem cell data	ISO 8472-1:2024 - Data interoperability for stem cell data — Part 1: Framework	Published (July 2024)
	ISO/DIS 8472-2 - Data interoperability for stem cell data — Part 2: Key characteristics of stem cell data	Under development
	ISO/AWI 8472-3 - Data interoperability for stem cell data — Part 3: Schema of the database structure	Under development
