# Supplementary material for: Promoting the adoption of best practices and standards to enhance quality and reproducibility of stem cell research
Source: Stem Cell Reports. 2025 Jun 12;20(7):102531. doi: 10.1016/j.stemcr.2025.102531 (PMC12277813; doi:10.1016/j.stemcr.2025.102531)
Supplement: Document S2. Article plus supplemental information [file mmc2.pdf]

Stem Cell Reports

Perspective

OPEN ACCESS

Promoting the adoption of best practices and standards to enhance quality and reproducibility of stem cell research

Lucia Selfa Aspiroz,[1,15,*] Milena Mennecozzi,[1,15] Laura Batlle,[2] Barbara Corneo,[3] Lyn Healy,[4] Mark Kotter,[5] Andreas Kurtz,[6] Tenneille E. Ludwig,[7,8] Christine Mummery,[9] Martin Pera,[10] Glyn N. Stacey,[11,12,13] Carlos A. Tristan,[14] and Maurice Whelan[1]

[1]European Commission, Joint Research Centre (JRC), Ispra, Italy
[2]Tissue Engineering Unit, Center for Genomic Regulation (CRG), Barcelona, Spain
[3]Stem Cell Core, Columbia University, New York, NY, USA
[4]Human Embryo and Stem Cell Unit, Human Biology Facility, The Francis Crick Institute, London, UK
[5]University of Cambridge and bit.bio Ltd, Cambridge, UK
[6]Berlin Institute of Health at Charite, Center for Regenerative Therapies, Berlin, Germany
[7]WiCell Research Institute, Madison, WI, USA
[8]Stem Cell and Regenerative Medicine Center, University of Wisconsin-Madison, Madison, WI, USA
[9]Department of Anatomy and Embryology, Institute of Human Organ and Disease Model Technologies (hDMT), Dept of Anatomy and Embryology, Leiden University Medical Center, Leiden, the Netherlands
[10]The Jackson Laboratory, Bar Harbor, ME USA
[11]International Stem Cell Biobanking Initiative, Barley, Hertfordshire, UK
[12]National Stem Cell Resource Centre, Institute of Zoology, Chinese Academy of Sciences, Beijing, China
[13]Beijing Institute for Stem Cell and Regenerative Medicine, Beijing, China
[14]National Center for Advancing Translational Sciences (NCATS), Stem Cell Translation Laboratory (SCTL), National Institutes of Health (NIH), Rockville, MD, USA
[15]These authors contributed equally
*Correspondence: idoia-lucia.selfa-aspiroz@ec.europa.eu
https://doi.org/10.1016/j.stemcr.2025.102531

SUMMARY

Advancing the use of human stem cell-based models on preclinical and regulatory testing fields requires the performance of rigorous and reproducible research. Quality standards and reporting best practices should be promoted to ensure the reliability and translatability of stem cell models and results. Strategies to increase awareness and implementation of best practices and standards will require training initiatives and collaboration across relevant stakeholders. Overall, improving the quality and reproducibility of stem cell-based models and methods through best practices and standards will accelerate their adoption in industrial and regulatory contexts and ultimately drive the development of effective therapies and safer chemicals.

INTRODUCTION

Human stem cell-based models are emerging as promising tools that could revolutionize drug discovery and safety assessment. They offer a powerful platform to increase the human relevance of efficacy and safety assessments in the development of pharmaceuticals, novel food and feed products, agrochemicals, cosmetics, and consumer goods (Suter-Dick et al., 2015; Blum et al., 2023). Traditionally, these industries have heavily relied on animal models, as reflected in most international guidelines for regulatory risk assessment, e.g., International Council for Harmonisation (ICH) S1B(R1) for carcinogenicity of pharmaceuticals (ICH, 2023), and Organization for Economic Cooperation and Development (OECD) Test guideline 415 for one-generation reproductive toxicity testing (OECD, 1983). However, due to ethical concerns, high costs, limited translatability of preclinical studies to humans, as well as societal and governmental pressure, there is growing interest in developing and implementing alternatives to animal experiments (European Parliament, 2021; European Citizens Initiative, 2023; European Commission, 2023).

Pharmaceutical companies are increasingly using new human *in vitro* models in their internal decision-making processes, and regulatory authorities are becoming more open to these alternative models. This shift is exemplified by the recent passage of the Food and Drug Administration Modernization Act 2.0 in the US (Pognan et al., 2023; Stresser et al., 2023). Additionally, international organizations such as the ICH and the OECD have launched initiatives to modernize their testing strategies, expanding the possibility of incorporating emerging human *in vitro* models. For example, the ICH S5 (R3) Guideline on detection of reproductive and developmental toxicity for human pharmaceuticals has been reviewed, and its updated version includes an annex with indications for the qualification of alternative assays for prediction of malformation or embryo-fetal lethality (ICH, 2020). Similarly, the OECD has published the Initial Recommendations on Evaluation of Data from the Developmental Neurotoxicity *In Vitro* Testing Battery, which summarizes the ongoing international efforts to move away from *in vivo* testing for identifying developmental neurotoxic chemicals (OECD, 2023).

© 2025 The Authors.
Published by Elsevier Inc. on behalf of International Society for Stem Cell Research.
This is an open access article under the CC BY license (http://creativecommons.org/licenses/by/4.0/).

Human induced pluripotent stem cells (hiPSCs) offer a highly valuable cellular source for generating physiologically relevant human models for both basic research and applied fields like toxicology and pharmacology (Shi et al., 2017). Thanks to the development of reprogramming protocols, adult somatic cells can regain pluripotency and turn into hiPSCs, providing a virtually unlimited source of any human differentiated cell type in the laboratory (Takahashi et al., 2007). In addition, the derivation of hiPSCs from somatic cells, rather than embryos, circumvents ethical concerns associated with the use of human embryonic stem cells (hESCs) and allows the generation of starting material from both healthy donors and patients, facilitating the development of novel *in vitro* disease models.

Despite the promise of hiPSC-based models, significant concerns remain regarding the rigor and reproducibility of biological research based on cell cultures. The Reproducibility Project: Cancer Biology underscored this issue, as researchers were only able to replicate the methodology of 50 out of the 193 planned experiments, 35 of which were *in vitro* studies (Errington et al., 2021). Numerous factors can contribute to reproducibility problems, including errors in study design, selective reporting practices, misidentification and contamination of research materials (e.g., cells, antibodies, and nucleic acid sequences), cellular decay over passages, and biological variability.

Biological variability in hiPSC-derived models has been linked to factors such as cell line selection, genetic background, genomic instability, differentiation protocols, and inaccurate standard operating procedures (SOPs) (Anderson et al., 2021). Multi-site studies have further demonstrated that, even when these factors are controlled, variability remains high and is related to local laboratory practices (Volpato et al., 2018).

The financial impact of irreproducibility in preclinical research alone was estimated as $28 billion annually (Freedman et al., 2015a), and even larger sums have been attributed to the use of unauthenticated cell lines (Harbut et al., 2024). While there is no single solution, it is essential to address these issues at early research stages as irreproducibility has multiple negative consequences, including erroneous data in the literature that can confuse and hinder scientific progress, waste resources, and erode public trust in scientific research.

Here, we explore the critical role of standardization in addressing reproducibility and advancing the field of human stem cell research. We provide an overview of current best practices and standards for cell culture methods and highlight strategies to promote their implementation early in stem cell research. By advocating for their implementation, we can significantly enhance the reproducibility and translational potential of human stem cell-based models and facilitate their uptake in industrial and regulatory sectors.

CURRENT BEST PRACTICES AND STANDARDS FOR STEM CELL RESEARCH

One effective strategy to address reproducibility challenges in stem cell research is the development and implementation of robust best practices and standards. Many advancements would not have been possible without the adoption of international standards. According to the International Organization for Standardization (ISO), a standard is a document, established by consensus and approved by a recognized body, that provides guidelines, specifications, or requirements to ensure that materials, products, processes, and services are fit for their intended purpose and consistently meet quality, safety, and efficiency criteria. Standards are primarily developed with industry in mind, as they aim to set consistent benchmarks for business and consumers, allowing interoperability within and between sectors. For instance, in the early days of the internet, organizations like World Wide Web Consortium and the Internet Engineering Task Force successfully led major global stakeholders toward adopting common standards for essential infrastructure components, resulting in significant long-term benefits. This demonstrates the importance of involving all relevant stakeholders in the development and adoption of standards within life sciences (GBSI, 2013). Regarding emerging technologies, standards can help in advancing scientific research discoveries to commercial products and clinical applications. Conformance to high-quality standards provides assurance to stakeholders on the quality and performance of the products, promoting their uptake by industrial end users and their acceptance in regulatory contexts of use (Piergiovanni et al., 2021).

Standards can be valuable for balancing the inherent biological variability of stem cells with the need for reproducible test results. Recently, several organizations have contributed to the development of documents outlining best practices and standards that are relevant to ensure the reproducibility and quality of stem cell research.

ISO has published formal standards applicable to cell culture and some specific to human stem cells (Table S1). The development of these standards is based on pre-normative work that identifies and documents the best practices agreed and shared between researchers. For example, ISO 24603:2022, which specifies the requirements for the biobanking of human and mouse pluripotent stem cells including collection of biological material, establishment, characterization, and quality control, is based on previous work of the International Stem Cell Banking Initiative

Table 1. Comparison of hPSC characterization requirements between ISO standards and best practices

	ISO 24603:2022	ISSCR Standards (2023)
Information of source cells (donor/patient)	x	x
Sex, age, ethnicity, health status, and tissue/cell type of origin	x	x
Cell line unique ID (hPSCreg)	x	x
Microbiological testing	x	x
Sterility testing (bacterial/fungus)	x	x
Mycoplasma testing	x	x
Infectious agents	x	x
Basic characterization		
Cell line identification/authentication	x (ISO/TS 23511)	x
Cell viability	x (ISO 23033:2021)	–
Cell morphology	x	–
Reprogramming factor elimination	x	x
Pluripotency characterization	x	x
Undifferentiated state markers (OCT4, NANOG, etc.)	x	x
Pluripotency assessment	x	x
In vitro differentiation (germ layer derivatives)	x	x
Teratoma assay	x	x
Genomic characterization	x	x
Assessment of genetic stability (karyotype, SNPs, WGS, etc.)	x	x
Timing of characterizations	–	x
Establishment of Quality Control Acceptance Criteria	x	–

(ISCBI, 2009) (Table 1). When appropriate and required, ISO expert working groups are formed, the community of stakeholders is consulted, and an international standard is developed and published to address specific technology and sector needs.

Moreover, best practices for cellular research have been developed to help the community incorporate them into their routine processes. The Guidance Document on Good Cell and Tissue Culture Practice (GCCP), published by the European Commission Joint Research Centre (JRC), aimed to establish principles for standardizing and harmonizing cell and tissue culture laboratory practices internationally. The first version, published in 2005, was updated in 2021 to incorporate advances in "omics" technology, stem cell research, and cell and tissue culture technologies (e.g., bioprinting, organ-on-chip) (Coecke et al., 2005; Pamies, 2021). This guidance promotes best practices in all aspects of cell and tissue use, including characterization and maintenance of essential properties, quality management, documentation and reporting, safety, education and training, and ethical considerations.

On the other hand, the OECD's Guidance Document on Good *In Vitro* Method Practices (GIVIMP), published in 2018, focuses on the procedures related to the development and implementation of cell- and tissue-based *in vitro* methods for regulatory use in human safety assessments (OECD, 2018). This document includes the GCCP principles and expands on requirements for facilities, instruments, test substances, quality management, use of SOPs, method performance criteria, data reporting, and data storage. These practices should be applied to ensure that the data generated with an *in vitro* method (e.g., stem cell-based method used to identify developmental neurotoxicity (OECD, 2023)) are of high quality, especially concerning reproducibility and accuracy, and can be used for decision-making, whether it is part of industrial internal evaluation or at regulatory level.

Lastly, the International Society for Stem Cell Research (ISSCR) Standards Initiative has recently published the Standards for Human Stem Cell Use in Research (Ludwig et al., 2023). Building upon the foundational work of the International Stem Cell Forum (www.stemcellforum.org), the International Stem Cell Initiative (The Steering Committee of the International Stem Cell Initiative, 2005), and the ISCBI (ISCBI, 2009; Stacey and Healy, 2021), the ISSCR Standards outline a set of recommendations establishing minimum characterization and reporting criteria for working with human stem cells in basic research. This document reflects the outcome of an international task force of scientists working toward a consensus on best practices that are both financially feasible and technically achievable by any laboratory. This means that no specific assays for characterization are recommended, but rather the currently available and most common assays are listed in the annexes (e.g., markers for monitoring differentiation, methods for genetic assessment).

Overall, given that standards are based on community best practices, there is broad overlap and many similarities among some of the documents mentioned earlier. For example, considering that both ISO 24603:2022 and the ISSCR Standards for Human Stem Cell Use in Research were developed building upon previous common recommendations (ISCBI, 2009), the minimum requirements for hPSC characterization are mostly shared. Differences mainly rely on the inclusion of timings for the characterizations in the ISSCR Standards document and the requirement for Quality Control Acceptance Criteria in the ISO standard. Additionally, checking cell morphology and cell viability are included in the basic characterization of the ISO standard (Table 1). Therefore, promoting a broad adoption of best practices such as the ISSCR Standards document early in basic research laboratories will strengthen the confidence in the reliability and quality of stem cell research outcomes and can help to speed up the uptake of reliable stem cell products in industrial and regulatory settings (Figure 1).

STANDARDS IMPLEMENTATION: STATE OF PLAY

Standards and best practices documents represent valuable tools for improving the rigor and reproducibility of stem cell research. However, the extent to which they are adopted by the relevant stakeholders remains uncertain, particularly since they were only recently published.

Adherence to ISO standards can impose a financial burden, as it involves both the cost of acquiring the relevant documents (which can range from EUR70 to EUR165 in the case of ISO Biotechnology standards) and the expense associated with implementing necessary changes. To demonstrate compliance to ISO standards, a certification process is required, where an independent body states that the product, service, or system is meeting the specific ISO requirements. In certain industries, ISO certification can help to demonstrate compliance to necessary regulatory requirements.

The adoption of quality and reporting standards has demonstrated an impact on basic research at different levels. In 2013, *Nature Research* journals implemented a mandatory reporting checklist to address the issues in the reliability and reproducibility of published research. This measure was followed by a 2017 survey of authors, who had published in *Nature* journals, which revealed that the reporting checklist had a significant impact on the reporting of statistics (Nature Editorial, 2018). In addition, majority of respondents stated that they continued using the checklist in their own work independently of the planned journal for publication (Nature Editorial, 2018). Another example of the impact of standards implementation was the reduction of genomic variability followed by the implementation of a quality management system based on ISO 9001:2015 and GIVIMP (Molina-Ruiz et al., 2022). Molina-Ruiz et al. showed that standardizing cell culture conditions and implementing genomic monitoring routinely can greatly improve genomic stability in hiPSCs, supporting the critical role of standards implementation to improve stem cell research reproducibility and quality.

To promote the adoption of best practices, the ISSCR developed a reporting checklist along with the Standards for Human Stem Cell Use in Research (https://www.isscr.org/basic-research-standards). This checklist intends to help in the preparation and assessment of manuscripts, ensuring the inclusion of critical details related to characterization, culture, experimental details, and data management in human stem cell research.

STRATEGIES TO PROMOTE STANDARDIZATION

Identifying potential barriers to adoption of best practices and standards can shed light on possible measures to promote and encourage their endorsement. Lessons learned from the open science initiatives, which aim to foster a research culture centered on transparency, openness, and reproducibility, demonstrate that promoting the implementation of new initiatives requires a cultural shift. Therefore, one of the main barriers to behavior change toward adoption of standards lies in the existing research culture. A comprehensive strategy for change has to address norms, incentives, and policies. Following best practices and standards to ensure the rigor and reproducibility should become the norm in the stem cell community. To achieve

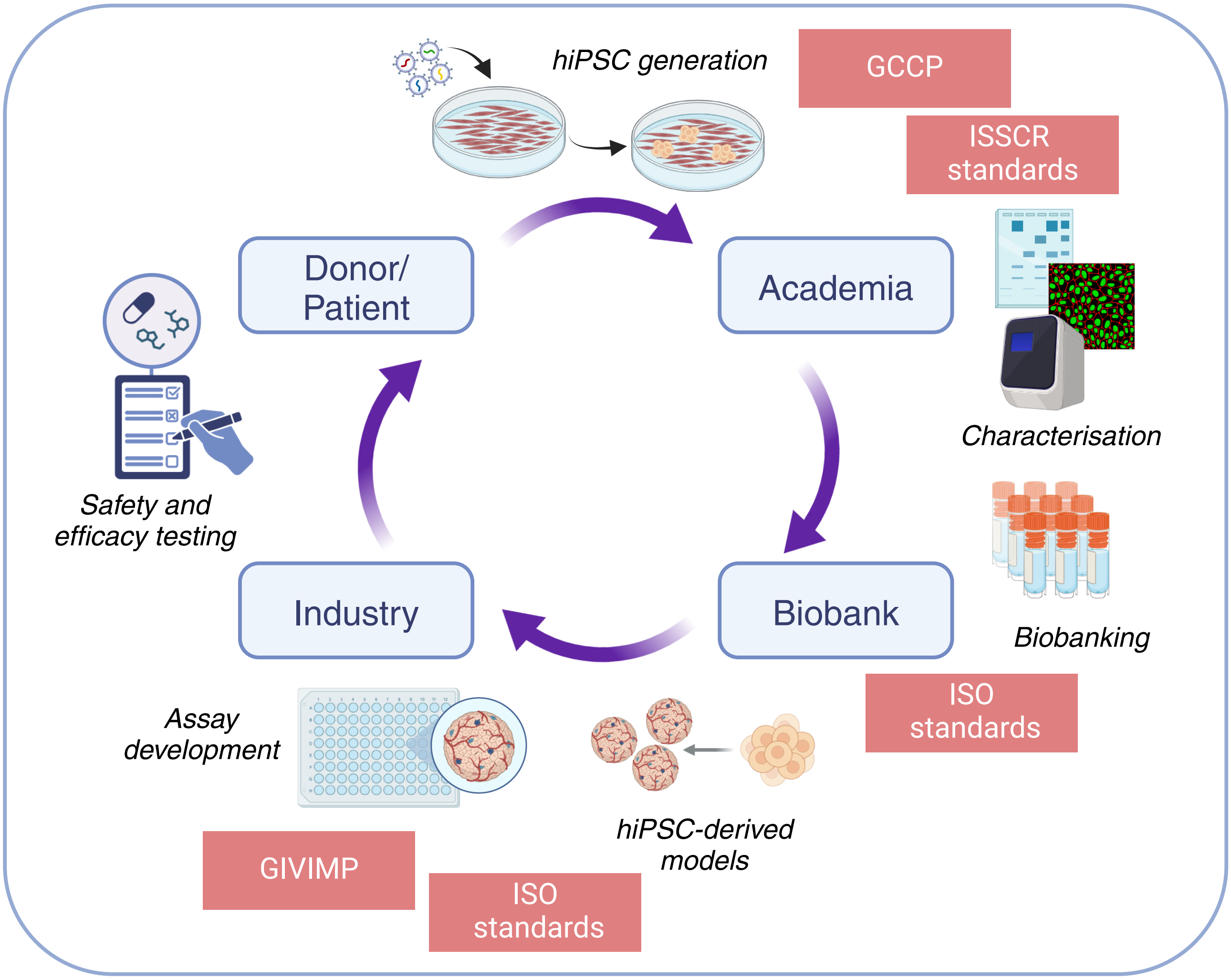

Figure 1. Conceptual representation of the R&D process of a stem cell-based method for efficacy and safety testing
hiPSCs are potentially generated from donor/patient samples in an academic research lab. After proper characterization, this new cell line can be deposited in a biobank. In an industrial R&D context, the cell line can be used to develop an hiPSC-derived model for safety and efficacy testing of a new therapeutic, advancing the development of novel treatments. To promote and expedite the translation process from academic to industrial context, different standards and best practices can be applied. GCCP: Good Cell Culture Practices; hiPSC: human induced pluripotent stem cell; ISSCR: International Society for Stem Cell Research; ISO: International Standard Organization; GIVIMP: Good *In Vitro* Method Practices. Created in BioRender. Selfa, L. (2025) https://BioRender.com/n68u032.

this, limited awareness of the existing guidelines and standards should be addressed, and the examples of best practice should become visible. For example, to promote the pre-registration of studies within the open research initiatives, scientific journals are adopting "badges" to acknowledge compliance of authors with this practice (Kidwell et al., 2016). Additionally, given that standards are followed on a voluntary basis, incentives and policy interventions need to take place to encourage their adoption. Open science has been included in different funding and publishing policies (Nosek et al., 2015; European Commission, 2021), and its practice should be rewarded in the researcher career progression, both at the level of awarding research grants and in recruitment processes (European Commission, 2017; Colavizza et al., 2020). Lastly, although implementing standards may require increased workload and resources, it should be weighed against the substantial time and financial losses associated with irreproducible experiments in both academic and industrial settings (Freedman et al., 2015a). Overall, defining strategic interventions addressing awareness, incentives, and resources will be essential to foster a research culture

where adherence to best practices and standards becomes the norm, ensuring reproducible and high-quality research outcomes in the stem cell field.

A multi-stakeholder approach to enhancing reproducibility of stem cell research

To achieve broader adoption of standards and enhance the reproducibility and quality of stem cell research, a multifaceted approach involving collaboration among different stakeholders is essential.

Research publishers, scientific journal editors, and manuscript reviewers can play a pivotal role in enforcing quality and reporting standards. Journal policies may include requirements for following specific guidelines or standards. For instance, *Cell Press* decided to improve reporting practices by transforming the Methods section of scientific publications. In the new STAR (Structured, Transparent, Accessible Reporting) Methods section, the experimental design and materials used in the study are described in high detail following a defined guideline, promoting transparency, accessibility, and reproducibility (Marcus, 2016). In the case of *in vitro* and cell line studies, the guideline requests reporting of culture conditions, the sex of the cells, and information on cell authentication (if available). On the other hand, the journal *Stem Cell Research* requires the completion of a pre-submission checklist for "Lab Resources" articles describing newly generated pluripotent stem cells (https://www.sciencedirect.com/journal/stem-cell-research/about/lab-resources/submit-your-lab-resource-article). This checklist includes requirements for cell line registration and unique cell identifier creation, cell line authentication, mycoplasma contamination testing, assessment of pluripotency, and genetic stability. This aligns with the ISSCR Standards Initiative, whose reporting practices checklist can be adopted by peer-reviewed journals to increase transparency in the reporting and implementation of best practices and key quality control measures in stem cell research (ISSCR, 2023). The journal *Stem Cell Reports* conducted a 6-month trial starting on 1st October 2023, requiring the use of the ISSCR Standards reporting checklist as part of the submission process for studies involving human stem cells. Analysis of the submissions during this trial revealed that there is still room for improvement in both the adoption and reporting of best practices in this field. Notably, a minority of manuscripts reported results addressing key standards criteria outlined in the checklist (Martin Pera, personal communication). The goal of this initial trial phase was to gather information and assess overall authors' compliance with the standards in the reporting list (Pera, 2023). Other journals, such as *Development*, have already expressed support for the ISSCR Standards Initiative by encouraging authors to reference the ISSCR reporting checklist during experimental design and manuscript preparation (Briscoe and Brown, 2024). Although editors may fear that a mandatory requirement could be a deterrent to publication in a journal, the goal of the reporting checklist is to raise awareness about the importance of implementing standards to achieve high-quality and reproducible research outputs in stem cell research. Manuscript reviewers are also essential to ensure adherence to established best practices and standards. While some journals may not enforce implementation of standards reporting checklists, peer reviewers can request such details to help them judge if the claims of the manuscript are supported by the quality of the biological material or if clarification is needed to confirm the validity or robustness of the research.

Research funding institutions should also consider developing and implementing new policies to foster reproducibility through standards adoption. The National Institutes of Health (NIH) announced the Rigor and Reproducibility policy in 2016 and revised grant application requirements to encourage scientific integrity, public accountability, and social responsibility in conducting research (https://grants.nih.gov/policy-and-compliance/policy-topics/reproducibility/guidance). A crucial component of this policy emphasizes the importance of authenticating key biological and/or chemical resources, including cell lines. Misidentification and cross-contamination of cell line cultures have been identified as significant sources of irreproducibility in biomedical sciences (Souren et al., 2022). Addressing this issue can be achieved through cell authentication practices, which involve determining and comparing the genetic signatures with established databases to confirm identity. Short tandem repeat (STR) profiling is the standardized method for authentication of human cells, although other DNA sequence-based methods, such as single-nucleotide polymorphism (SNP) profiling, can also be used (Almeida et al., 2016). STR authentication service provided by third parties is relatively low cost, typically ranging from $65 to $200 per sample (Harbut et al., 2024). On the other hand, the European Commission requires grantees to register their hESC and hiPSC lines in the hPSCreg registry, attesting their ethical provenance and biological potency and creating a unique identifier (Kurtz et al., 2022). The impact of neglecting authentication practices in stem cell research was highlighted in a study where STR testing of commercial hiPSC-derived cardiomyocytes after completing experiments revealed that one of the cell products was derived from a human embryonic stem cell instead of an hiPSC line (Gintant et al., 2020). By including cell identification, authentication, and other quality control measures in grant requirements and providing additional financial support when needed, research funding organizations can increase awareness and incentivize the broader adoption of

best practices in cell culture work. To promote the adoption of best practices, research funding institutions could incorporate compliance with reporting standards checklists into their funding renewal processes. Making continued financial support dependent on adherence to reporting standards would promote accountability, increase awareness, and ensure consistent application of best practices throughout the project.

Core facilities are well positioned to contribute to the promotion of best practices and standards adoption. They provide access to expensive, state-of-the-art technologies and advanced skills in fields like microscopy and stem cell culture in a cost- and time-effective manner. Core facilities also generate large amounts of research resources and data (Kos-Braun et al., 2020). In the stem cell research field, core facilities within and across countries are uniting efforts to harmonize methodologies and quality control measures, aiming to increase the overall reproducibility of the results produced in different laboratories (CorEuStem, www.coreustem.eu and Stem Cell COREdinates, www.coredinates.org) (Dahéron et al., 2021). Core facilities offer training in best cell culture practice and quality control of hiPSC to investigators new to the field, many of whom lack expertise in basic or applied stem cell biology. However, most stem cell core facilities operate on a fee-for-service business model, making it difficult to secure funding for continuous education of core personnel in new techniques and best practices. Funding mechanisms that specifically support stem cell core facilities could alleviate the financial burden and enable them to better support research and innovation. For example, the Netherlands Organization for Scientific Research has set a National Roadmap for Large-Scale Research Infrastructure, which recently allocated EUR13.5 million to establish hDMT INFRA StemCells (www.hdmt.technology/2023/02/20/13-5-million-euros-for-hdmt-infra-stemcells/). This will be a national large-scale facility to support setting up and conducting stem cell studies, increasing reliability through standardization and automation, and providing hands-on training. Programs from other research fields, such as the funding call launched by the Chan Zuckerberg Initiative to directly support the work of imaging scientists employed in imaging core facilities at non-profit universities or research institutes across the world (https://chanzuckerberg.com/rfa/chan-zuckerberg-initiative-imaging-scientists/), could be translated for stem cell core facilities.

In addition to following existing best practices and adopting quality and reporting standards, complementary approaches may be necessary to improve reproducibility and comparability of results across laboratories. In the field of hiPSC research, variables such as donor-to-donor differences, genetic stability, and differentiation protocols can significantly impact the reproducibility of the experiments and models derived. Acknowledging this variability should be accompanied by defining strategies to minimize it (Volpato and Webber, 2020). Rigorous quality control measures for cell identity, genetic stability, undifferentiated state and pluripotency assessment, and mycoplasma testing should be implemented during the generation of hiPSC lines, as it is done in the generation of large hiPSC panels in reputable stem cell banks such as EBiSC (www.ebisc.org) and WiCell (www.wicell.org) (De Sousa et al., 2017; Huang et al., 2019; Steeg et al., 2021). The storage and distribution of well-characterized cell lines performed by these platforms also enable broader access to high-quality starting material and resources generated and used by research projects worldwide.

Ultimately, education and training in all stages of research careers are crucial for increasing awareness and fostering adoption of best practices and standards (Freedman et al., 2015b; Souren et al., 2022). The JRC of the European Commission is deeply involved in several education and training activities, including the organization, since 2017, of the JRC Summer School to share knowledge and experience on human relevant cutting-edge technologies, including induced pluripotent stem cells (iPSCs), organ-on-chip, and computational modeling (https://joint-research-centre.ec.europa.eu/events/jrc-summer-school-non-animal-approaches-science-2025-05-19_en). Similarly, the European Organ-on-Chip Society (EUROoCS) has been hosting the EUROoCS Summer School since 2023, which offers lectures and hands-on sessions on topics like stem cells, organoids, GCCP, and regulatory processes (https://euroocs.eu/summer-school/). Aspects of best practices and reproducibility for emerging technologies are covered in the training program of both summer schools to raise awareness among early-career scientists. Free online educational resources are also available to provide support for researchers involved in *in vitro* test method development interested in ensuring the quality and reproducibility of their technologies, including training modules on GIVIMP (https://learn.etplas.eu/courses/eu-60/). By investing in training programs and promoting a culture of reproducibility, we can collectively improve the quality and reliability of stem cell research.

Establishing reference cell lines and databases to enhance reproducibility

The introduction of agreed reference material provides another opportunity to promote reproducibility. For instance, monoclonal antibody reference standards have been introduced to minimize the inherent variability across different assays (WHO, 2022). However, cells are significantly more complex than antibodies, and therefore, standardization is challenging. There is ongoing discussion about the utility of establishing and using reference hiPSC

lines, also referred to as Rosetta lines. These lines are defined as hiPSC lines commonly used across multiple laboratories, enabling researchers to address experimental variation (Volpato and Webber, 2020). One example is WTSIi018-B-12 (KOLF2.1J), a proposed reference line generated within the iPSC Neurodegenerative Disease Initiative from the NIH's Center for Alzheimer's and Related Dementias. Its robustness as a reference line was demonstrated by benchmarking its differentiation potential to several cell types using 10 different protocols and comparing to 12 other hiPSC lines in independent laboratories (Pantazis et al., 2022). However, it is important to recognize that multiple reference lines will be needed to better represent population genetic diversity, including lines from different sexes and diverse genetic ancestries. Furthermore, the risks of cell line cross-contamination, culture mislabeling, and emergence of genetic variants mean that such lines must be carefully controlled and a common source reference stock of the original cell line must be retained. Nevertheless, there are further opportunities for defining quality attributes of hiPSC-derived cells, such as the definition of minimum cell characteristics (e.g., marker genes, functional properties).

Creating comprehensive reference databases documenting cell line derivation, characterization, and experimental conditions can further enable benchmarking and comparability across studies. To create such databases, cell lines must be associated with unique, persistent, and findable cell line identifiers through registration linked to data in interoperable formats (Wells et al., 2024). A unique cell line identifier helps researchers to gather and link all data associated with a specific line. This allows effectively connecting the physical cell entity to its digital representation and facilitates the assessment of reproducibility between laboratories using the same cell line. As biological entities are dynamic by nature, their digital phenotypes are also dynamic. Their dynamic nature can be related to a reference point, for example, by providing a persistent identifier to a static digital phenotype, as practiced in hPSCreg (www.hpscreg.eu). In practice, a unique identifier is linked to a pluripotent stem cell phenotype determined at the time of identifier registration. It is essential to monitor genetic changes in culture and confirm that the cells are still fit for purpose. If identified genetic changes are stable, the new phenotype can be registered as a subclone of a parent with a new digital identifier, making phenotypic dynamics traceable.

Defining standard criteria for the description of cell lines ("what" data should be recorded and registered) and agreeing on using machine-readable metadata format ("how" data should be recorded and registered) are critical to provide tools for data exchange and comparison within the stem cell community. By promoting Findable, Accessible, Interoperable, and Reusable) data management practices (Wilkinson et al., 2016), stem cell researchers can contribute to a more open, transparent, and collaborative research environment, ultimately enhancing the impact and reproducibility of their work.

It is important to consider that the quality of research is ultimately in the hands of researchers. Their awareness of how established standards are directly relevant to their work, along with their continued participation in ongoing discussions about standards and best practices as these technologies evolve, will be essential toward enhancing and maintaining reproducibility and rigor in the stem cell field. This is encouraged by a number of collaborative and community-driven activities in the stem cell field including the ISCBI (www.iscbi.org), the ISSCR (www.isscr.org), CorEuStem (www.coreustem.eu), and Stem Cell COREdinates (https://coredinates.org/).

CONCLUSION

Human stem cell research has the potential to revolutionize preclinical and regulatory safety testing by providing more human relevant models that can accelerate the development of life-saving cures and protect citizens from harmful chemicals. However, the translation potential of academic stem cell research can be hindered by the variability and irreproducibility inherent to *in vitro* research. Therefore, promoting the implementation of quality and reporting standards needs to start at the level of basic research and involve the collaboration and communication among all relevant stakeholders. While standards implementation can be deemed as costly, it is essential to balance these costs with the benefits in terms of robustness and reproducibility. Additionally, supporting funding should be contemplated as a potentially additional driver of standardization.

Overall, working on specific actions to increase awareness and incentivize the implementation of best practices and standards requires a cultural shift toward prioritizing quality and reproducibility. To collectively work on implementation of stem cell standards, we propose the following recommendations.

(1) Broader inclusion of quality and reporting standards related to human stem cell research in scientific journal policies.
(2) Establish research funder policies requiring quality and reporting standards, such as cell line authentication, identification, and registration.
(3) Support and fund stem cell core facilities, key players in implementing and generating standardized materials and procedures.
(4) Gain community consensus on the establishment of reference cell lines and databases for data exchange

and comparison, and on the definition of the different iPSC-derived cell type's core characteristics.
(5) Prioritize quality of starting material by promoting the use of cell lines from reputable suppliers such as stem cell biobanks.
(6) Establish and improve training programs to increase awareness and proper implementation of existing best practices and standards.

Proactive engagement will be key to the success of these initiatives to collectively improve the rigor and reproducibility of stem cell research. This, in turn, will potentially enhance the adoption of stem cell-based models and methods and boost innovation in drug discovery and regulatory safety assessments.

DECLARATION OF INTERESTS

C.M. is an associate editor of *Stem Cell Reports*. M.P. is an associate editor of *Stem Cell Reports* and is a member of the International Stem Cell Initiative. C.M. and M.P. receive compensation for services as editors of the journal.

SUPPLEMENTAL INFORMATION

Supplemental information can be found online at https://doi.org/10.1016/j.stemcr.2025.102531.

REFERENCES

Almeida, J.L., Cole, K.D., and Plant, A.L. (2016). Standards for Cell Line Authentication and Beyond. PLoS Biol. *14*, e1002476. https://doi.org/10.1371/journal.pbio.1002476.

Anderson, N.C., Chen, P.F., Meganathan, K., Afshar Saber, W., Petersen, A.J., Bhattacharyya, A., Kroll, K.L., and Sahin, M.; Cross-IDDRC Human Stem Cell Working Group (2021). Balancing serendipity and reproducibility: Pluripotent stem cells as experimental systems for intellectual and developmental disorders. Stem Cell Rep. *16*, 1446–1457. https://doi.org/10.1016/j.stemcr.2021.03.025.

Blum, J., Masjosthusmann, S., Bartmann, K., Bendt, F., Dolde, X., Dönmez, A., Förster, N., Holzer, A.K., Hübenthal, U., Keßel, H.E., et al. (2023). Establishment of a human cell-based in vitro battery to assess developmental neurotoxicity hazard of chemicals. Chemosphere *311*, 137035. https://doi.org/10.1016/j.chemosphere.2022.137035.

Briscoe, J., and Brown, K. (2024). Publishing in Development: integrity, transparency and accessibility. Development *151*, dev204202. https://doi.org/10.1242/dev.204202.

Coecke, S., Balls, M., Bowe, G., Davis, J., Gstraunthaler, G., Hartung, T., Hay, R., Merten, O.W., Price, A., Schechtman, L., et al. (2005). Guidance on Good Cell Culture Practice: A Report of the Second ECVAM Task Force on Good Cell Culture Practice. Altern. Lab. Anim. *33*, 261–287. https://doi.org/10.1177/026119290503300313.

Colavizza, G., Hrynaszkiewicz, I., Staden, I., Whitaker, K., and McGillivray, B. (2020). The citation advantage of linking publications to research data. J.M. Wicherts, ed. *15*, e0230416. https://doi.org/10.1371/journal.pone.0230416.

Dahéron, L., Diecke, S., Healy, L., and D'Souza, S. (2021). Cores laboratories: Organization for stem cell technology advancement. Stem Cell Res. *53*, 102266. https://doi.org/10.1016/j.scr.2021.102266.

De Sousa, P.A., Steeg, R., Wachter, E., Bruce, K., King, J., Hoeve, M., Khadun, S., McConnachie, G., Holder, J., Kurtz, A., et al. (2017). Rapid establishment of the European Bank for induced Pluripotent Stem Cells (EBiSC) - the Hot Start experience. Stem Cell Res. *20*, 105–114. https://doi.org/10.1016/j.scr.2017.03.002.

Errington, T.M., Mathur, M., Soderberg, C.K., Denis, A., Perfito, N., Iorns, E., and Nosek, B.A. (2021). Investigating the replicability of preclinical cancer biology. eLife *10*, e71601. https://doi.org/10.7554/eLife.71601.

European Citizens Initiative (2023). Save cruelty-free cosmetics — Commit to a Europe without animal testing. https://citizens-initiative.europa.eu/initiatives/details/2021/000006_en.

European Commission (2017). Evaluation of Research Careers Fully Acknowledging Open Science Practices: Rewards, Incentives And/or Recognition for Researchers Practicing Open Science (LU: Publications Office). https://data.europa.eu/doi/10.2777/75255.

European Commission (2021). Horizon Europe, Open Science: Early Knowledge and Data Sharing, and Open Collaboration (LU: Publications Office). https://data.europa.eu/doi/10.2777/18252.

European Commission (2023). Communication from the Commission on the European Citizens' Initiative (ECI) 'Save cruelty-free cosmetics — Commit to a Europe without animal testing'. https://single-market-economy.ec.europa.eu/publications/communication-commission-european-citizens-initiative-eci-save-cruelty-free-cosmetics-commit-europe_en.

European Parliament (2021). MEPs demand EU action plan to end the use of animals in research and testing. https://www.europarl.europa.eu/news/en/press-room/20210910IPR11926/meps-demand-eu-action-plan-to-end-the-use-of-animals-in-research-and-testing.

Freedman, L.P., Cockburn, I.M., and Simcoe, T.S. (2015a). The Economics of Reproducibility in Preclinical Research. PLoS Biol. *13*, e1002165. https://doi.org/10.1371/journal.pbio.1002165.

Freedman, L.P., Gibson, M.C., Ethier, S.P., Soule, H.R., Neve, R.M., and Reid, Y.A. (2015b). Reproducibility: changing the policies and culture of cell line authentication. Nat. Methods *12*, 493–497. https://doi.org/10.1038/nmeth.3403.

GBSI (2013). The Case for Standards in Life Science Research: Seizing Opportunities at a Time of Critical Need (Washington D.C.: Global Biological Standards Institute). https://scholar.google.com/scholar_lookup?title=The%20Case%20for%20Standards%20in%20Life%20Science%20Research:%20Seizing%20Opportunities%20at%20a%20Time%20of%20Critical%20Need&publication_year=2013&.

Gintant, G., Kaushik, E.P., Feaster, T., Stoelzle-Feix, S., Kanda, Y., Osada, T., Smith, G., Czysz, K., Kettenhofen, R., Lu, H.R., et al. (2020). Repolarization studies using human stem cell-derived cardiomyocytes: Validation studies and best practice recommendations. Regul. Toxicol. Pharmacol. *117*, 104756. https://doi.org/10.1016/j.yrtph.2020.104756.

Harbut, E., Makris, Y., Pertsemlidis, A., and Bleris, L. (2024). The history, landscape, and outlook of human cell line authentication and security. SLAS Discov. *29*, 100194. https://doi.org/10.1016/j.slasd.2024.100194.

Huang, C.-Y., Liu, C.L., Ting, C.Y., Chiu, Y.T., Cheng, Y.C., Nicholson, M.W., and Hsieh, P.C.H. (2019). Human iPSC banking: barriers and opportunities. J. Biomed. Sci. *26*, 87. https://doi.org/10.1186/s12929-019-0578-x.

ICH (2020). ICH S5 (R3) Guideline on detection of reproductive and developmental toxicity for human pharmaceuticals. https://www.ema.europa.eu/en/ich-s5-r3-guideline-detection-reproductive-developmental-toxicity-human-pharmaceuticals-scientific-guideline.

ICH (2023). ICH S1B(R1) Guideline on testing for carcinogenicity of pharmaceuticals. https://www.ema.europa.eu/en/ich-guideline-s1br1-testing-carcinogenicity-pharmaceuticals-scientific-guideline.

International Stem Cell Banking Initiative (2009). Consensus Guidance for Banking and Supply of Human Embryonic Stem Cell Lines for Research Purposes. Stem Cell Rev. Rep. *5*, 301–314. https://doi.org/10.1007/s12015-009-9085-x.

ISSCR Task Force for Basic Research Standards Electronic address tludwig@wicell org (2023). SnapShot: Reporting practices for publishing results with human PSCs and tissue stem cells. Stem Cell Rep. *18*, 1898–1898.e1. https://doi.org/10.1016/j.stemcr.2023.08.010.

Kidwell, M.C., Lazarević, L.B., Baranski, E., Hardwicke, T.E., Piechowski, S., Falkenberg, L.S., Kennett, C., Slowik, A., Sonnleitner, C., Hess-Holden, C., et al. (2016). Badges to Acknowledge Open Practices: A Simple, Low-Cost, Effective Method for Increasing Transparency. M.R. Macleod, ed. *14*, e1002456. https://doi.org/10.1371/journal.pbio.1002456.

Kos-Braun, I.C., Gerlach, B., and Pitzer, C. (2020). A survey of research quality in core facilities. eLife *9*, e62212. https://doi.org/10.7554/eLife.62212.

Kurtz, A., Mah, N., Chen, Y., Fuhr, A., Kobold, S., Seltmann, S., and Müller, S.C. (2022). Human pluripotent stem cell registry: Operations, role and current directions. Cell Prolif. *55*, e13238. https://doi.org/10.1111/cpr.13238.

Ludwig, T.E., Andrews, P.W., Barbaric, I., Benvenisty, N., Bhattacharyya, A., Crook, J.M., Daheron, L.M., Draper, J.S., Healy, L.E., Huch, M., et al. (2023). ISSCR standards for the use of human stem cells in basic research. Stem Cell Rep. *18*, 1744–1752. https://doi.org/10.1016/j.stemcr.2023.08.003.

Marcus, E.; whole Cell team (2016). A STAR Is Born. Cell *166*, 1059–1060. https://doi.org/10.1016/j.cell.2016.08.021.

Molina-Ruiz, F.J., Introna, C., Bombau, G., Galofre, M., and Canals, J.M. (2022). Standardization of Cell Culture Conditions and Routine Genomic Screening under a Quality Management System Leads to Reduced Genomic Instability in hPSCs. Cells *11*, 1984. https://doi.org/10.3390/cells11131984.

Nature Editorial (2018). Checklists work to improve science. Nature *556*, 273–274. https://doi.org/10.1038/d41586-018-04590-7.

Nosek, B.A., Alter, G., Banks, G.C., Borsboom, D., Bowman, S.D., Breckler, S.J., Buck, S., Chambers, C.D., Chin, G., Christensen, G., et al. (2015). Promoting an open research culture. Science *348*, 1422–1425. https://doi.org/10.1126/science.aab2374.

OECD (1983). Test No. 415: One-Generation Reproduction Toxicity Study. OECD (OECD Guidelines for the Testing of Chemicals, Section 4). https://www.oecd.org/en/publications/test-no-415-one-generation-reproduction-toxicity-study_9789264070844-en.html.

OECD (2018). Guidance Document on Good In Vitro Method Practices (GIVIMP). OECD. (OECD Series on Testing and Assessment). https://www.oecd.org/en/publications/guidance-document-on-good-in-vitro-method-practices-givimp_9789264304796-en.html.

OECD (2023). Initial Recommendations on Evaluation of Data from the Developmental Neurotoxicity (DNT) In-Vitro Testing Battery. https://one.oecd.org/document/ENV/CBC/MONO(2023)13/en/pdf.

Pamies, D., Leist, M., Coecke, S., Bowe, G., Allen, D.G., Gstraunthaler, G., Bal-Price, A., Pistollato, F., de Vries, R.B.M., Hogberg, H.T., et al. (2022). Guidance document on Good Cell and Tissue Culture Practice 2.0 (GCCP 2.0)'. ALTEX *39*, 30–70. https://doi.org/10.14573/altex.2111011.

Pantazis, C.B., Yang, A., Lara, E., McDonough, J.A., Blauwendraat, C., Peng, L., Oguro, H., Kanaujiya, J., Zou, J., Sebesta, D., et al. (2022). A reference human induced pluripotent stem cell line for large-scale collaborative studies. Cell Stem Cell *29*, 1685–1702.e22. https://doi.org/10.1016/j.stem.2022.11.004.

Pera, M.F. (2023). Achieving the goals of the ISSCR standards initiative. Stem Cell Rep. *18*, 1743. https://doi.org/10.1016/j.stemcr.2023.08.009.

Piergiovanni, M., Leite, S.B., Corvi, R., and Whelan, M. (2021). Standardisation needs for organ on chip devices. Lab Chip *21*, 2857–2868. https://doi.org/10.1039/D1LC00241D.

Pognan, F., Beilmann, M., Boonen, H.C.M., Czich, A., Dear, G., Hewitt, P., Mow, T., Oinonen, T., Roth, A., Steger-Hartmann, T., et al. (2023). The evolving role of investigative toxicology in the pharmaceutical industry. Nat. Rev. Drug Discov. *22*, 317–335. https://doi.org/10.1038/s41573-022-00633-x.

Shi, Y., Inoue, H., Wu, J.C., and Yamanaka, S. (2017). Induced pluripotent stem cell technology: a decade of progress. Nat. Rev. Drug Discov. *16*, 115–130. https://doi.org/10.1038/nrd.2016.245.

Souren, N.Y., Fusenig, N.E., Heck, S., Dirks, W.G., Capes-Davis, A., Bianchini, F., and Plass, C. (2022). Cell line authentication: a necessity for reproducible biomedical research. EMBO J. *41*, e111307. https://doi.org/10.15252/embj.2022111307.

Stacey, G.N., and Healy, L. (2021). The International Stem Cell Banking Initiative (ISCBI). Stem Cell Res. *53*, 102265. https://doi.org/10.1016/j.scr.2021.102265.

Steeg, R., Mueller, S.C., Mah, N., Holst, B., Cabrera-Socorro, A., Stacey, G.N., De Sousa, P.A., Courtney, A., and Zimmermann, H. (2021). EBiSC best practice: How to ensure optimal generation, qualification, and distribution of iPSC lines. Stem Cell Rep. *16*, 1853–1867. https://doi.org/10.1016/j.stemcr.2021.07.009.

Stresser, D.M., Kopec, A.K., Hewitt, P., Hardwick, R.N., Van Vleet, T. R., Mahalingaiah, P.K.S., O'Connell, D., Jenkins, G.J., David, R., Graham, J., et al. (2024). Towards in vitro models for reducing or

replacing the use of animals in drug testing. Nat. Biomed. Eng. *8*, 930–935. https://doi.org/10.1038/s41551-023-01154-7.

Suter-Dick, L., Alves, P.M., Blaauboer, B.J., Bremm, K.D., Brito, C., Coecke, S., Flick, B., Fowler, P., Hescheler, J., Ingelman-Sundberg, M., et al. (2015). Stem Cell-Derived Systems in Toxicology Assessment. Stem Cells Dev. *24*, 1284–1296. https://doi.org/10.1089/scd.2014.0540.

Takahashi, K., Tanabe, K., Ohnuki, M., Narita, M., Ichisaka, T., Tomoda, K., and Yamanaka, S. (2007). Induction of Pluripotent Stem Cells from Adult Human Fibroblasts by Defined Factors. Cell *131*, 861–872. https://doi.org/10.1016/j.cell.2007.11.019.

The Steering Committee of the International Stem Cell Initiative (2005). The International Stem Cell Initiative: toward benchmarks for human embryonic stem cell research. Nat. Biotechnol. *23*, 795–797. https://doi.org/10.1038/nbt0705-795.

Volpato, V., Smith, J., Sandor, C., Ried, J.S., Baud, A., Handel, A., Newey, S.E., Wessely, F., Attar, M., Whiteley, E., et al. (2018). Reproducibility of Molecular Phenotypes after Long-Term Differentiation to Human iPSC-Derived Neurons: A Multi-Site Omics Study. Stem Cell Rep. *11*, 897–911. https://doi.org/10.1016/j.stemcr.2018.08.013.

Volpato, V., and Webber, C. (2020). Addressing variability in iPSC-derived models of human disease: guidelines to promote reproducibility. Dis. Model. Mech. *13*, dmm042317. https://doi.org/10.1242/dmm.042317.

Wells, C.A., Guhr, A., Bairoch, A., Chen, Y., Hu, M., Löser, P., Ludwig, T.E., Mah, N., Mueller, S.C., Seiler Wulczyn, A.E.M., et al. (2024). Guidelines for managing and using the digital phenotypes of pluripotent stem cell lines. Stem Cell Rep. *19*, 1369–1378. https://doi.org/10.1016/j.stemcr.2024.08.009.

WHO (2022). WHO manual for the preparation of reference materials for use as secondary standards in antibody testing. https://www.who.int/publications/m/item/who-manual-for-reference-material-for-antibody-testing.

Wilkinson, M.D., Dumontier, M., Aalbersberg, I.J.J., Appleton, G., Axton, M., Baak, A., Blomberg, N., Boiten, J.W., da Silva Santos, L. B., Bourne, P.E., et al. (2016). The FAIR Guiding Principles for scientific data management and stewardship. Sci. Data *3*, 160018. https://doi.org/10.1038/sdata.2016.18.

Stem Cell Reports, Volume *20*

Supplemental Information

Promoting the adoption of best practices and standards to enhance quality and reproducibility of stem cell research

Lucia Selfa Aspiroz, Milena Mennecozzi, Laura Batlle, Barbara Corneo, Lyn Healy, Mark Kotter, Andreas Kurtz, Tenneille E. Ludwig, Christine Mummery, Martin Pera, Glyn N. Stacey, Carlos A. Tristan, and Maurice Whelan

Supplemental Information

Supplementary Table 1: ISO Biotechnology Standards relevant to stem cell culture

General topic	ISO reference	Status
Analytical methods (cell line testing and characterisation)	ISO/TS 23511:2023 - General requirements and considerations for cell line authentication	Published (May 2023)
	ISO/CD 23511 - General requirements and considerations for cell line authentication	Under development, will replace ISO/TS 23511:2023
	ISO 24479:2024 - Cellular morphological analysis – General requirements and considerations for cell morphometry to quantify cell morphological features	Published (October 2024)
	ISO/DIS 8934-1 - Cell viability analytical methods- Part 1: General requirements and considerations	Under development
	ISO 20391:2018 - Cell counting - Part 1: General guidance on cell counting methods	Published (January 2018)
	ISO/CD 20391-1 - Cell counting - Part 1: General guidance on cell counting methods	Under development, will replace ISO 20391-1:2018
	ISO 20391-2:2019 - Cell counting - Part 2: Experimental design and statistical analysis to quantify counting methods	Published (August 2019)
	ISO 24190:2023 - Analytical methods – Risk-based approach for method selection and validation for rapid microbial detection in bioprocesses	Published (May 2023)
	ISO/CD TR 4752 - Inventory of methods for detection of microbiological contamination in mammalian cell culture	Under development
Biobanking	ISO 21709:2020 - Biobanking – Process and quality requirements for establishment, maintenance and characterisation of mammalian cell lines	Published with an amendment ISO 21709:2020/Amd 1:2021
	ISO 24603:2022 - Biobanking - Requirements for human and mouse pluripotent stem cells	Published (August 2022)

	ISO 18162:2024 - Biobanking - Requirements for human neural stem cells derived from pluripotent stem cells	Published (December 2024)
	ISO/DIS 20012 - Biobanking - Requirements for human natural Killer cells derived from pluripotent stem cells	Under development
	ISO/TS 22859:2022 - Biobanking – Requirements for human mesenchymal stromal cells derived from umbilical cord tissue	Published (July 2022)
	ISO 24651:2022 - Biobanking - Requirements for human mesenchymal stromal cells derived from bone marrow	Published (August 2022)
Data interoperability for stem cell data	ISO 8472-1:2024 - Data interoperability for stem cell data — Part 1: Framework	Published (July 2024)
	ISO/DIS 8472-2 - Data interoperability for stem cell data — Part 2: Key characteristics of stem cell data	Under development
	ISO/AWI 8472-3 - Data interoperability for stem cell data — Part 3: Schema of the database structure	Under development
